# Emerging Roles for Hox Proteins in the Last Steps of Neuronal Development in Worms, Flies, and Mice

**DOI:** 10.3389/fnins.2021.801791

**Published:** 2022-02-04

**Authors:** Weidong Feng, Yinan Li, Paschalis Kratsios

**Affiliations:** ^1^Department of Neurobiology, University of Chicago, Chicago, IL, United States; ^2^University of Chicago Neuroscience Institute, Chicago, IL, United States; ^3^Committee on Development, Regeneration, and Stem Cell Biology, University of Chicago, Chicago, IL, United States; ^4^Committee on Neurobiology, University of Chicago, Chicago, IL, United States

**Keywords:** neuronal development, terminal identity, Hox genes, transcription factors, terminal selectors, synapse formation, synapse maturation

## Abstract

A remarkable diversity of cell types characterizes every animal nervous system. Previous studies provided important insights into how neurons commit to a particular fate, migrate to the right place and form precise axodendritic patterns. However, the mechanisms controlling later steps of neuronal development remain poorly understood. Hox proteins represent a conserved family of homeodomain transcription factors with well-established roles in anterior-posterior (A-P) patterning and the early steps of nervous system development, including progenitor cell specification, neuronal migration, cell survival, axon guidance and dendrite morphogenesis. This review highlights recent studies in *Caenorhabditis elegans*, *Drosophila melanogaster* and mice that suggest new roles for Hox proteins in processes occurring during later steps of neuronal development, such as synapse formation and acquisition of neuronal terminal identity features (e.g., expression of ion channels, neurotransmitter receptors, and neuropeptides). Moreover, we focus on exciting findings suggesting Hox proteins are required to maintain synaptic structures and neuronal terminal identity during post-embryonic life. Altogether, these studies, in three model systems, support the hypothesis that certain Hox proteins are continuously required, from early development throughout post-embryonic life, to build and maintain a functional nervous system, significantly expanding their functional repertoire beyond the control of early A-P patterning.

## Introduction

Nervous system development is a multi-step process that generates a multitude of cell types. Dividing progenitor cells, or neural stem cells, will ultimately give rise to distinct types of neurons and glia. Newly born, post-mitotic neurons face a number of early challenges before participating into a functional neural circuit. They need to be molecularly specified, migrate to the right place, and acquire distinct axo-dendritic morphologies. Studies in all major model organisms suggest that these early steps of nervous system development are often controlled by Hox proteins, a conserved family of homeodomain transcription factors critical for anterior-posterior (A-P) patterning and formation of the animal body plan. The roles of Hox proteins during the early steps of nervous system development have been summarized in excellent reviews (Di Bonito et al., 2013a; Philippidou and Dasen, 2013; Parker and Krumlauf, 2020). Here, we highlight recent studies in *Caenorhabditis elegans*, *Drosophila melanogaster*, and mice that uncovered new roles for Hox in the last steps of neuronal development. We define as “last steps” the processes of synapse formation and acquisition of neuronal terminal identity features (e.g., expression of neurotransmitter [NT] receptors, neuropeptides, ion channels) because such processes represent the final events that lead to the establishment of a functional neural circuit. Perhaps more strikingly, a number of Hox proteins are continuously expressed in post-mitotic neurons of invertebrate and vertebrate nervous systems (discussed herein). Depletion of Hox gene activity at later stages of development and post-embryonic life supports the emerging hypothesis that Hox proteins are required not only to establish, but also maintain synaptic structures and terminal identity features. This review will focus on these exciting studies, offering new insights into the function of Hox proteins in the final steps of neuronal development.

## The Role of Hox Genes in Late Stages of *Caenorhabditis elegans* Nervous System Development

**FIGURE 1:**
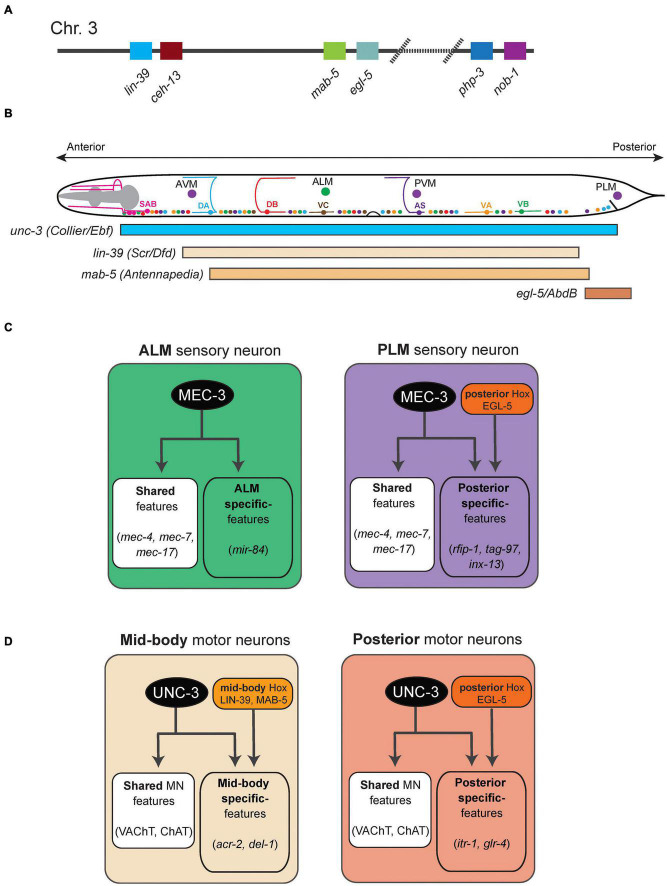
Hox gene functions in mechanosensory and motor neurons in *C. elegans*. **(A)** Schematic of the *C. elegans* Hox gene cluster. **(B)** Schematic showing the cell body location of mechanosensory neurons (AVM, ALM, PVM, PLM) and cholinergic MNs in the ventral nerve cord (SAB, DA, DB, VA, VB, VC, AS). The GABAergic MNs are not shown. **(C)** The terminal selector MEC-3 controls ALM and PLM terminal identity. The activity of the posterior Hox gene *egl-5* diversifies PLM from ALM. Examples of terminal identity genes are shown in italics. **(D)** An intersectional strategy for the control of terminal identity of midbody (UNC-3, LIN-39, MAB-5) and posterior (UNC-3, EGL-5) MNs along the A-P axis of the *C. elegans* ventral nerve cord. See text for details.

The *C. elegans* genome contains six Hox genes. The anterior Hox (*ceh-13/Lab/Hox1*) together with the mid-body Hox (*lin-39/Scr/Dfd/Hox3-5 and mab-5/Antp/Hox6-8*) and posterior Hox (*egl-5/AbdB/Hox9-13*) genes were identified 30 years ago (Costa et al., 1988; Clark et al., 1993; Van Auken et al., 2000), whereas two additional posterior Hox genes (*nob-1*, *php-3*) were discovered in 2000 (Van Auken et al., 2000). *C. elegans* Hox genes are organized in three different sub-clusters located on Chromosome III (Figure 1A). Previous work revealed that *C. elegans* Hox genes control A-P patterning and the development of lateral epidermis and ventral ectoderm (Kenyon, 1986; Costa et al., 1988; Chisholm, 1991; Cowing and Kenyon, 1992; Clark et al., 1993; Wang et al., 1993; Wittmann et al., 1997; Brunschwig et al., 1999). Critical roles for Hox genes have also been described during the early steps of *C. elegans* nervous system development, that is, in the specification and survival of neuronal progenitors (Fixsen et al., 1985; Kenyon, 1986; Clark et al., 1993; Salser et al., 1993; Wang et al., 1993; Kalis et al., 2014), cell migration (Chisholm, 1991; Salser and Kenyon, 1992; Harris et al., 1996; Sym et al., 1999; Tihanyi et al., 2010; Wang et al., 2013), and neurite/axonal growth (Jia and Emmons, 2006; Zheng et al., 2015a). Below, we focus on recent studies uncovering functions for *C. elegans* Hox genes in processes occurring during later stages of neuronal development, such as synapse formation and acquisition of terminal identity.

### Control of Synapse Formation/Maturation in *Caenorhabditis elegans*

The posterior Hox gene *egl-5* is necessary for migration of the hermaphrodite-specific motor neuron (HSN) from the tail to the vulva, where it stimulates vulva muscle contraction resulting in egg laying (Baum et al., 1999). In posteriorly located sensory neurons, *egl-5* controls neurite outgrowth (Zheng et al., 2015a). Besides its involvement in cell migration and neurite outgrowth, *egl-5* also controls the wiring of the posteriorly located cholinergic motor neuron DA9 (Kratsios et al., 2017). In wild-type animals, the DA9 axon extends circumferentially to reach the dorsal body wall muscle and form *en passant* neuromuscular synapses. In *egl-5* mutants, these DA9 synapses are generated at the “wrong” place; they are found more anteriorly when compared to wild-type animals, suggesting a synaptic specificity defect. Interestingly, split GFP reporter technology (GRASP) also revealed that the DA9 synaptic input (received by the AVG interneuron) fails to be maintained in adult *egl-5* mutants, despite being properly established at earlier larval stages, indicating a critical role for *egl-5* in synapse maintenance. Together, these findings suggest that the posterior Hox gene *egl-5* controls both synaptic input and output of a posterior cholinergic motor neuron (DA9) in *C. elegans*.

### Control of Neuronal Terminal Identity by *Caenorhabditis elegans* Hox Genes

Once post-mitotic neurons have established synapses, the function of every neuronal circuit critically relies on the ability of its constituent neurons to communicate with each other via neurotransmitters and/or neuropeptides, as well as to display neuron type-specific electrophysiological signatures. These function-defining features are determined by the expression of neurotransmitter (NT) biosynthesis proteins, ion channels, neuropeptides, NT receptors, and cell adhesion molecules. Genes coding for such proteins have been termed “terminal identity” genes (Hobert, 2008; Hobert and Kratsios, 2019), and are expressed continuously – from late developmental stages through adulthood – to determine the final (mature) identity and function of each neuron type. Recent studies on two different neuron types in *C. elegans*, namely the touch receptors and nerve cord motor neurons, revealed a new role for Hox genes in the control of neuronal terminal identity (Table 1). We highlight these studies below.

**TABLE 1:** Hox gene studies focused on late steps of nervous system development.

Species	Gene	Description	References
*C. elegans*	*egl-5*	*egl-5* regulates terminal differentiation of PLM	Toker et al., 2003
*C. elegans*	*egl-5*	EGL-5 is required for subtype-specific circuit formation by acting in both the sensory neuron and downstream interneuron to promote functional connectivity in touch receptor neurons.	Zheng et al., 2015a
*C. elegans*	*ceh-13*	CEH-13 functions cell non-autonomously to guide ALM migration and axonal outgrowth	Zheng et al., 2015a
*C. elegans*	*php-3*	PHP-3 makes PLM neurons morphologically distinct from ALM neurons independently with egl-5	Zheng et al., 2015a
*C. elegans*	*nob-1*	*nob-1* is needed to generate the cells that become the PLM neurons	Zheng et al., 2015b
*C. elegans*	*ceh-13, egl-5*	CEH-13 and EGL-5 act as transcriptional guarantors to ensure reliable and robust *mec-3* expression during terminal neuronal differentiation of touch receptor neurons	Zheng et al., 2015b
*C. elegans*	*lin-39, mab-5, egl-5*	Hox genes function as *unc-3* co-factors to specify cholinergic motor neuron sub-class terminal identities	Kratsios et al., 2017
*C. elegans*	*lin-39, mab-5*	*lin-39* and *mab-5* regulates and maintains subtype specific terminal identities of both cholinergic and GABAergic motor neurons	Feng et al., 2020
*C. elegans*	*lin-39 mab-5*	Hox genes regulates *cfi-1* to regulate ventral cord motor neuron terminal identity	Li et al., 2020
*C. elegans*	*egl-5*	*egl-5* is crucial for HSN to adopt the serotonergic identity	Chisholm, 1991
*C. elegans*	*egl-5*	*egl-5* regulates HSN terminal identity through regulating UNC-86	Baum et al., 1999
*C. elegans*	*egl-5*	*egl-5* is required for the adoption of dopaminergic identity for ray cells through regulation of dbl-1	Lints and Emmons, 1999
*C. elegans*	*ceh-13*	*ceh-13* specifies the terminal identity of two GABAergic motor neurons DD1 and DD2	Aquino-Nunez et al., 2020
*Drosophila*	*Ubx*	*Ubx* acts in both muscles and motoneurons to orchestrate formation of specific neuromuscular connections	Hessinger et al., 2017
*Drosophila*	*Abd-B*	Temporal control of neuronal differentiation by *Abd-B* in the context of CCAP peptidergic neurons	Moris-Sanz et al., 2015
*Drosophila*	*Ubx, abd-A*	Segmentally homologous neurons acquire two different terminal neuropeptidergic fates in the Drosophila nervous system	Gabilondo et al., 2018
*Drosophila*	*Ubx, abd-A*	*Ubx* and *abd-A* are required to maintain the expression of the neuropeptide *Lk* in larval stages	Estacio-Gomez et al., 2013
*Drosophila*	*Dfd*	*Dfd* is continuously required to maintain the expression of Ankyrin2 extra large (Ank2-XL) and thus synaptic stability in head motor neurons (MNs) that innervate the mouth hood elevator (MHE) and depressor (MHD) muscles	Friedrich et al., 2016
Mouse	*Hoxa2*	Hoxa2-dependent development of the mouse facial somatosensory map	Oury et al., 2006
Mouse	*Hoxa2*	Hoxa2 selects barrelette neuron identity and connectivity in the mouse somatosensory brainstem.	Bechara et al., 2015
Mouse	*Hox2*	Hox2 genes are required for tonotopic map precision and sound discrimination in the mouse auditory brainstem	Karmakar et al., 2017
Mouse	*Hoxa5*	Hoxa5 functions early after birth to impact expression of genes with synaptic function	Lizen et al., 2017b
Mouse	*Hoxa5*	Hoxa5 specifies pontine neuron positional identity and input connectivity	Maheshwari et al., 2020
Mouse	*Hoxc8*	*Hoxc8* is required for the maintenance of terminal identity genes *Nrg1, Mcam*, and *Pappa* in spinal motor neurons.	Catela et al., 2021
Mouse	*Hox5*	Late removal of *Hox5* genes depletes PMC motor neuron number and branches, suggesting it is continuously required for the survival of these neurons.	Philippidou et al., 2012

#### Hox Genes Control Terminal Identity Features of *Caenorhabditis elegans* Touch Receptor Neurons

In *C. elegans*, there are six touch receptor neurons (TRNs) mediating sensory responses to light touch. TRNs are classified into four subtypes: (a) bilaterally symmetrical pairs of ALM and PLM neurons are located at the midbody and tail region, respectively and (b) single AVM and PVM neurons are located in the midbody (Figure 1B). ALM and PLM are born embryonically, while AVM and PVM are generated post-embryonically. TRNs synapse onto and provide input to command interneurons (PVC, AVB, AVD, AVA), which stimulate downstream motor neurons, thus generating touch reflex responses. Early specification and differentiation of TRNs have been well investigated (Bounoutas and Chalfie, 2007), but how each TRN subtype acquires its unique terminal identity remains poorly understood.

At the behavioral level, animals lacking *egl-5* (posterior Hox) gene activity are touch-insensitive at the tail, suggesting defects in the development of the posteriorly located PLM neuron (Chisholm, 1991). Later studies indeed demonstrated that *egl-5* is necessary for PLM terminal identity (Toker et al., 2003; Zheng et al., 2015a; Figure 1C). In addition to *egl-5*, two other poster Hox genes (*nob-1*, *php-3*) control PLM development; *nob-1* is necessary for the generation of PLM precursors, whereas *php-3* together with *egl-5* diversifies PLM from its more anteriorly located counterpart, the ALM neuron. Lastly, *egl-5* controls PLM morphological characteristics, such as neurite length, by repressing anterior Hox genes (*lin-39, mab-5*) and TALE cofactors (Zheng et al., 2015a). The case of *egl-5* highlights a recurring theme of Hox gene action across model systems, that is, Hox genes are required for various facets of development of a specific neuron type.

In the more anteriorly located ALM neurons, the anterior Hox gene *ceh-13* regulates ALM terminal identity, as evidenced by reduced expression of a handful of terminal identity genes (*mec-4, mec-7, mec-17, mec-18*) in *ceh-13* mutant animals. Mechanistically, these studies proposed that CEH-13 and EGL-5 function as transcriptional guarantors by controlling the levels of expression of the terminal selector gene *mec-3*, which in turn is required for terminal identity of both ALM and PLM neurons (Figure 1C; Zheng et al., 2015a,b). CEH-13 and EGL-5 increase the probability of *mec-3* transcriptional activation by the POU-homeodomain transcription factor UNC-86 via the same Hox/Pbx binding site in ALM and PLM neurons respectively. This molecular mechanism ensures robustness of TRN terminal differentiation.

A multifaceted role of Hox genes is evident during the development of the *C. elegans* touch-reflex circuit: (a) Hox genes are involved in both early (e.g., generation of TRN precursor cells) and late steps of TRN development (e.g., terminal identity). (b) All six *C. elegans* Hox genes affect TRN development in various ways: *ceh-13* regulates ALM terminal identity; *lin-39 and mab-5* regulate the migration of AVM/PVM precursor; *egl-5* and *php-3* regulate PLM terminal identity; *nob-1* is necessary for the generation of PLM precursors. (c) Although it remains mechanistically unclear how they control TRN-specific terminal identity genes (e.g., NT receptors, ion channels, neuropeptides), two Hox proteins (CEH-13, EGL-5) appear to act directly as transcriptional guarantors of *mec-3*, the terminal selector for all *C. elegans* TRNs. (d) Intriguingly, Hox genes control the development of neurons at different layers (sensory, interneuron) of the touch-reflex circuit. That is, sensory TRN terminal identity requires Hox gene function, whereas the identity of the PVC command interneurons (which receive sensory input from the PLM touch receptors) requires *egl-5* gene activity (Chisholm, 1991; Zheng et al., 2015a).

#### Hox Genes Control Terminal Identity Features of Ventral Nerve Cord Motor Neurons

Similar to the touch receptor studies described above, the availability of terminal identity markers for ventral nerve cord motor neurons (MNs) in *C. elegans* has critically advanced our mechanistic understanding of Hox gene function in the nervous system. Nine distinct classes of MNs are found in the nerve cord of *C. elegans* hermaphrodite animals. Based on neurotransmitter usage, they can be classified into two categories: cholinergic (SAB, DA, DB, VA, VB, AS, VC) and GABAergic (DD, VD) MNs (Figure 1B). The SAB, DA, DB, and DD classes are generated embryonically, whereas the VA, VB, VC, VD, and AS neurons are generated post-embryonically (Von Stetina et al., 2006). The terminal identity of most cholinergic MN classes in the nerve cord (SAB, DA, DB, VA, VB, AS) critically depends on the terminal selector UNC-3, member of the conserved family of Collier/Olf/Ebf(COE) family of TFs (Prasad et al., 1998, 2008; Kratsios et al., 2011, 2015). Mechanistically, UNC-3 binds directly to the *cis*-regulatory region of terminal identity genes (e.g., acetylcholine [ACh] biosynthesis proteins, ion channels, neuropeptides) and activates their transcription. The homeodomain TF UNC-30 (PITX) acts in an analogous manner in GABAergic (DD, VD) MNs (Jin et al., 1994; Eastman et al., 1999).

In the context of both cholinergic and GABAergic MNs, recent work demonstrated that Hox genes act as cofactors of terminal selectors (Kratsios et al., 2017; Feng et al., 2020). In GABAergic MNs, the mid-body Hox genes *lin-39* and *mab-5* collaborate with *unc-30* to control terminal identity gene expression. In cholinergic MNs, *lin-39* and *mab-5* collaborate with *unc-3* to activate expression of several terminal identity genes (*unc-129, del-1, acr-2, dbl-1, unc-77, slo-2*) (Figure 1D). Like UNC-3, chromatin immunoprecipitation experiments suggest that LIN-39 and MAB-5 act directly (Kratsios et al., 2017; Feng et al., 2020). Apart from this UNC-3 co-factor role, *lin-39* is also the rate-limiting factor for ensuring cholinergic MN identity. In the absence of *unc-3*, LIN-39 no longer binds to the *cis*-regulatory region of cholinergic MN genes. Instead, it relocates and switches targets, resulting in ectopic activation of alternative identity genes (Feng et al., 2020). Hence, the terminal selector UNC-3 prevents a Hox transcriptional switch to safeguard cholinergic MN identity.

Are Hox genes required during adulthood to maintain terminal identity features and thereby ensure continuous functionality of individual neuron types? Inducible, protein depletion experiments using the auxin inducible degradation (AID) system demonstrated that the midbody Hox protein LIN-39 is required in adult life to maintain MN terminal identity features (Feng et al., 2020; Li et al., 2020). This finding was somewhat unexpected because Hox genes are mostly thought to act early during animal development. Additional work on Hox is needed in *C. elegans* and other model systems to rigorously test whether maintenance of neuronal terminal identity is a key feature of Hox gene function in the nervous system.

The organization of cholinergic MNs into distinct subtypes along the A-P axis also offers an opportunity to dissect the molecular mechanisms underlying neuronal subtype identity. For example, the DA class of nine MNs can be subdivided into four subtypes based on cell boy position: DA1 is located at the anterior ganglion (retrovesicular ganglion [RVG]), DA2–7 are located at the VNC, and DA8–9 are found at the posterior ganglion (preanal ganglion [PAG]). In addition to their position, cholinergic MN subtypes do show distinct connectivity features and expression profiles of terminal identity genes (Kratsios et al., 2017). Hox genes control cholinergic MN subtype identity along the A-P axis of the *C. elegans* nervous system via an intersectional strategy that involves the terminal selector UNC-3 (Kratsios et al., 2017). For example, UNC-3 is expressed in all 9 DA neurons, but collaborates with the mid-body Hox genes *lin-39* and *mab-5* in mid-body DA2-7 neurons to control their terminal identity (Figure 1D). Similarly, UNC-3 and the posterior Hox gene *egl-5* determine posterior MN (DA9) terminal identity (Figure 1D). Although the molecular mechanism of *egl-5* activity in posterior MNs is unknown, biochemical evidence suggests that LIN-39 – like UNC-3 – acts directly by binding on the *cis*-regulatory region of terminal identity genes. This direct mode of regulation further extends to intermediary TFs (*cfi-1/Arid3a, bnc-1/Bnc1/2*) responsible for MN subtype identity (Kerk et al., 2017; Li et al., 2020).

The role of the anterior Hox gene *ceh-13* during *C. elegans* neuronal terminal differentiation is largely elusive, partly due to the early larval lethality of *ceh-13* mutants (Brunschwig et al., 1999). A recent study suggested *ceh-13* controls terminal identity features of GABAergic motor neurons (DD1, DD2) located in the anterior ganglion, but the underlying mechanisms remain unknown (Aquino-Nunez et al., 2020).

#### Posterior Hox Gene *egl-5* Controls the Identity of Serotonergic and Dopaminergic Neurons

In addition to its role on posterior MNs, the posterior Hox gene *egl-5* controls the terminal identity of two other neuron types. The hermaphrodite specific neurons (HSNs) partially lose their ability to produce serotonin in *egl-5* mutants (Chisholm, 1991). Moreover, *egl-5* acts in tail sensory neurons of the *C. elegans* male. Upon *egl-5* genetic removal, these neurons do not adopt dopaminergic fate and cannot be induced to express dopamine (Lints and Emmons, 1999).

## The Role of Hox Genes in Late Stages of *Drosophila* Nervous System Development

**FIGURE 2:**
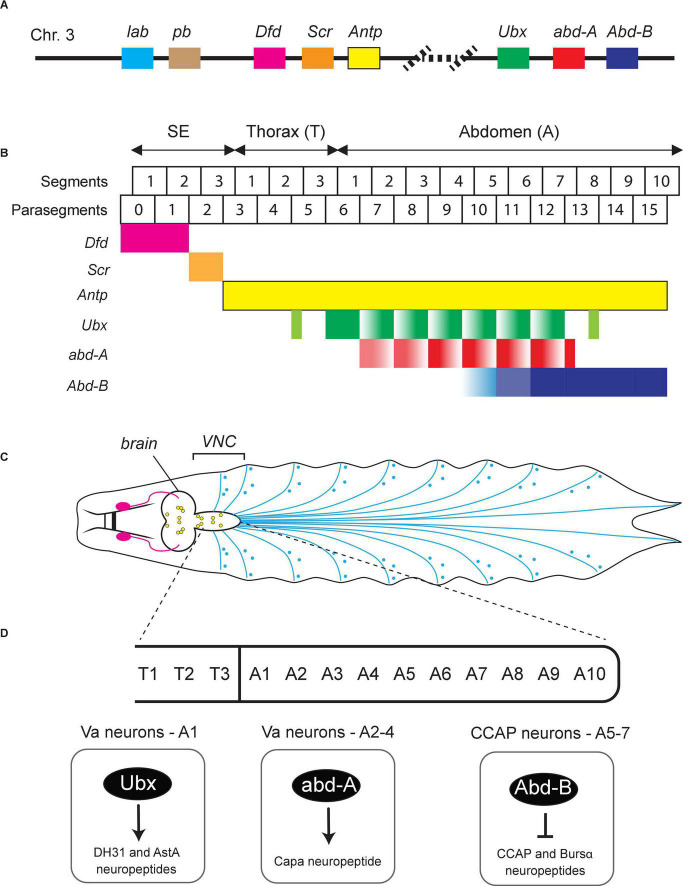
Hox gene expression in the *Drosophila* nerve cord. **(A)** Schematic of the *Drosophila* Hox gene cluster. **(B)** Six Hox genes (*Dfd, Scr, Antp, Ubx, abd-A, Abd-B*) are expressed in the *Drosophila* ventral nerve cord (VNC). Their expression pattern along the A-P axis is color-coded. SE, subesophagus; Th, thorax; Ab, abdomen. Adapted from Estacio-Gomez and Diaz-Benjumea (2014). **(C)** Schematic of the nervous system in *Drosophila* larvae showing the brain, VNC, and the peripheral nervous system at the late 3rd instar stage. Adapted from Sokabe et al. (2016). **(D)** Examples of *Drosophila* Hox genes that control terminal identity features of VNC neurons. See text for details.

Eight Hox genes are embedded in the genome of the fruit fly *Drosophila melanogaster*: *labial (lab), proboscipedia (pb), Deformed (Dfd), Sex combs reduced (Scr), Antennapedia (Antp), Ultrabithorax (Ubx), abdominal-A (abd-A*) and *Abdominal-B (Abd-B*) (Figure 2A). Hox genes were first discovered in *Drosophila* during the 20th century; genetic experiments identified mutants with dramatic phenotypes caused by homeotic transformations (e.g., legs instead of antennae in *Antp* mutants, duplication of thoracic segments in *Ubx* mutants) (Nusslein-Volhard and Wieschaus, 1980). Subsequent studies showed that the principles of Hox gene function and their role in establishing the body plan along the A-P axis are conserved across species.

Based on their chromosomal location, *Drosophila* Hox genes are organized in two gene complexes. The Antennapedia complex or Antp-C (consisting of *lab, pb, Dfd, Scr*, and *Antp*) specifies the anterior body plan from the head to the anterior thorax, while the Bithorax complex or Bx-C (consisting of *Ubx, abd-A*, and *Abd-B*) specifies the segments in posterior thorax and abdomen (Figure 2B). An important characteristic of Hox gene expression is their “temporal and spatial collinearity,” that is, the genes located at the 3′ end of a complex/cluster are expressed earlier and more rostrally than those residing at the 5′ end (Kmita and Duboule, 2003; Gaunt, 2015). This appears to be a highly conserved property of Hox genes and has been found in *Drosophila* and many other species (Gaunt, 2015).

During development, neurons in *Drosophila* arise from neuroblasts (NBs) located in three thoracic (T1–T3) and eight abdominal (A1–A8) segments of the ventral nerve cord (VNC) (Figures 2C,D). These NBs possess the potency to generate any neuron type, but they give rise to unique types of neuronal progenies depending on their location along the A-P axis. This spatial pattern of distinct neuronal types correlates with the combinatorial expression pattern of Hox genes along the A-P axis of the *Drosophila* body. During neurogenesis, Hox gene activity guides NBs to exit the cell cycle and promotes (or blocks) apoptosis, eventually leading to a spatial map of unique neuron types (Estacio-Gomez and Diaz-Benjumea, 2014; Gummalla et al., 2014). Moreover, *Drosophila* Hox genes control additional steps during early nervous system development, such as neuronal specification and axo-dendritic morphogenesis (Reichert and Bello, 2010; Baek et al., 2013; Estacio-Gomez and Diaz-Benjumea, 2014). We will discuss below recent studies suggesting Hox genes also control later steps of *Drosophila* nervous system development, such as synapse formation and neuronal terminal identity (Table 1).

### Control of Synapse Formation/Maturation by *Drosophila* Hox Genes

Compelling evidence suggests that the *Drosophila* Hox gene *Ubx* controls neuromuscular synapse formation in the embryo (Hessinger et al., 2017). Interestingly, it does so by acting both in muscles and motor neurons. In abdominal segments A1–A7 of wild-type embryos, RP motor neurons innervate the ventrolateral muscles VL1-4. However, these motor neurons fail to make correct contacts with muscle VL1 in *Ubx* mutant embryos. Mechanistically, this study provides an intriguing link between Hox and Wnt signaling pathway – Wnt is instrumental for neuromuscular synapse formation across species (Klassen and Shen, 2007; Strochlic et al., 2012; Kerr et al., 2014). The authors proposed a model in which Ubx controls, in VL2 muscles, *Wnt4* expression. Upon its secretion, Wnt4 is sensed by motor neurons (destined to innervate the VL1 muscles) via the Wnt receptor Fz-2. The Ubx-dependent Wnt4 signal from VL2 muscles triggers the repulsion of arriving growth cones belonging to motor neurons, hence these neurons innervate different muscles (VL1). Although the precise mechanism of Ubx function in these motor neurons remains obscure, rescue experiments clearly demonstrated that *Ubx* orchestrates the interaction between two cell types, muscles and motor neurons, to regulate the establishment of neuromuscular synapses in the fly embryo.

A second example of Hox gene involvement in *Drosophila* synapse formation comes from head motor neurons (MNs) that innervate the mouth hook elevator (MHE) and depressor (MHD) muscles, which coordinate the elevation and depression of the mouth hook (MH). The anterior Hox gene *Dfd* is expressed in a subset of MNs that specifically innervate the MHE (Friedrich et al., 2016). These *Dfd*-expressing MNs play a critical role in controlling the MH-dependent motor behaviors, including hatching at the end of embryogenesis and feeding in larval stages. In *Dfd* mutants, while the number of these MNs remains unchanged, they fail to extend axonal projections to their muscle targets, resulting in failure to hatch. Intriguingly, removing *Dfd* after the establishment of synaptic connections also results in impaired MH movements in larvae, suggesting *Dfd* is continuously required for the normal functions of these MNs (Friedrich et al., 2016). Genetically, *Dfd acts* upstream of a microtubule-organizing complex which is important for synapse stability even after their establishment. *Dfd* is continuously required to maintain the expression of Ankyrin2 extra large (Ank2-XL), which is known to be involved in determining the physical properties of synapses. Importantly, synaptic specificity is dependent on actions of *Dfd* both in motor neurons and muscles, reminiscent of the Ubx case discussed above (Hessinger et al., 2017). Altogether, the above studies on *Drosophila Ubx* and *Dfd* support the hypothesis that Hox genes, in addition to their well-documented roles in motor neuron specification, survival and axonal pathfinding (Baek et al., 2013; Philippidou and Dasen, 2013), also control the establishment and maintenance of neuromuscular synapses.

### Control of Neuronal Terminal Identity by *Drosophila* Hox Genes

Much of our current understanding of Hox gene function in the *Drosophila* nervous system derives from studies on the abdominal leucokinergic neurons (ABLKs), which express the neuropeptide *Leucokinin* (*Lk*) and are often used as a model system to study both embryonic and post-embryonic neurogenesis (Estacio-Gomez and Diaz-Benjumea, 2014). During embryonic neurogenesis, the NB5-5 progenitor gives rise to 7 pairs of embryonic ABLKs (eABLKs), one in each of the first 7 abdominal segments (A1–7) of the VNC (Figures 2B,C). *Lk* is not initially expressed in the eABLKs when they are born but becomes detectable at later developmental stages (first instar larva). Later, additional post-embryonic ABLKs (pABLKs) are generated (third instar larva), and express *Lk* in pupal stages. The cell type-specific expression of the terminal identity gene encoding *Lk* is critically dependent on Bx-C (*Ubx, abd-A, Abd-B*) gene activity (Estacio-Gomez et al., 2013). Although *Lk* is a single Hox-dependent terminal identity gene, this study does suggest a later role for Hox in *Drosophila* neurons.

Hox genes are also expressed in the neuroectoderm at early development, but then become silenced when NBs delaminate and are reactivated at later stages in specific neurons. The posterior abdominal Hox genes, *Ubx* and *abd-a*, are expressed in post-mitotic eABLKs in the first instar larvae, where they are redundantly required for the expression of *Lk*. Moreover, when both *Ubx* and *abd-A* are knocked down specifically from early second instar larvae, it results in loss of *Lk* expression in late third instar larvae (Estacio-Gomez et al., 2013), suggesting maintenance of *Lk* in eABLKs relies on continuous expression of *Ubx* and *abd-A*. On the other hand, the other posterior Hox gene *Abd-B* represses *Lk* expression in non-ABLK cells during both embryonic and larval neurogenesis. Similarly, *Abd-B* is continuously required to maintain the repression of *Lk*, as removing *Abd-B* from first instar larvae results in de-repression of *Lk* and increased number of ABLKs in third star larvae and adults. Another study on *Abd-B* yielded similar results in the context of the crustacean cardioactive peptide (CCAP)-expressing neurons, which control ecdysis (Moris-Sanz et al., 2015). In A5–7 segments, the CCAP efferent neurons are defined by the expression of two terminal identity markers – the neuropeptides CCAP and Bursicon α (Bursα). Using a hypomorphic allele, the authors found that Abd-B represses CCAP/Bursα in early larvae. Hence, the precise onset of CCAP/Bursα expression critically relies on Abd-B-mediated repression (Figure 2D). Moreover, RNAi-induced knocked down of *Abd-B* in the first instar larve results in expression of the CCAP/Bursα neuropeptides, suggesting Abd-B is continuously required to maintain repression of CCAP/Bursα (Moris-Sanz et al., 2015).

The Hox genes *Ubx* and *abd-A* are also necessary to diversify the terminal identity of distinct neuropeptidergic neurons in the first four abdominal (A1–A4) segments of the fly VNC (Gabilondo et al., 2018). In A1, *Ubx* controls the identity of ventral abdominal (Va) neurons expressing the neuropeptides DH31 and AstA (Figure 2D). In A2–A4, *abd-A* controls the identity of distinct Va neurons expressing the neuropeptide Capa (Figure 2D). The diversification of these neuropeptidergic neurons is a product of regionalized, segment-specific Hox gene expression. For example, *abd-A* is not expressed in A1, whereas *Ubx* is expressed in all abdominal segments. Regionalized expression in the nervous system is a common feature between invertebrate and vertebrate Hox genes. In particular, the case of Va neuropeptidergic neurons where segment-specific Hox genes control segment-specific Va neuron terminal identity is reminiscent of a Hox-based strategy used by *C. elegans* MNs. In that case, mid-body (*lin-39, mab-5*) and posterior (*egl-5*) Hox genes control the terminal identity of mid-body and posterior MNs, respectively (Figure 1D; Kratsios et al., 2017).

Although the underlying mechanisms remain unknown, these studies strongly suggest that Hox genes can establish and maintain terminal identity features of post-mitotic neurons in *Drosophila*.

## The Role of Hox Genes in Late Stages of Mouse Nervous System Development

**FIGURE 3:**
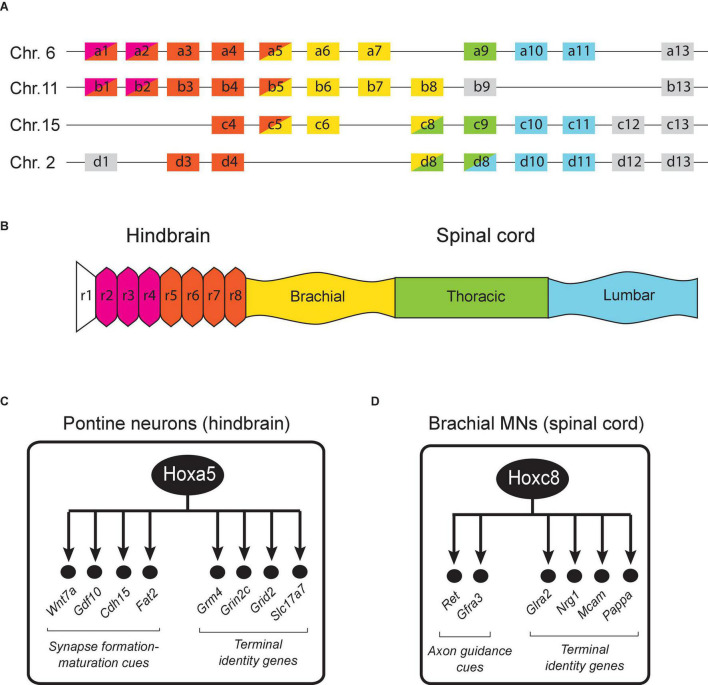
Hox gene expression in the mouse hindbrain and spinal cord. **(A)** The 39 Hox genes in mice are distributed in four clusters (a, b, c, d). **(B)** Region-specific Hox gene expression is shown along the rostrocaudal axis of the embryonic nervous system. Adapted from Philippidou and Dasen (2013). The dynamic nature of Hox gene expression is not illustrated for simplicity. **(C)** Schematic summary of putative Hoxa5 target genes in pontine neurons. See text for details. **(D)** Schematic summary of Hoxc8 target genes in brachial MNs. See text for details.

During early vertebrate evolution, the single Hox gene cluster of vertebrate ancestors was duplicated twice, eventually giving rise to four clusters – *HoxA, HoxB, HoxC*, and *HoxD* in mammals (Soshnikova et al., 2013). In mice, these four clusters contain 39 Hox genes, which are further categorized into 13 paralog groups (PG) based on their relative position within the clusters and gene sequence (Figure 3A). The majority of these Hox genes are expressed in the mouse central nervous system (CNS) during development (Krumlauf et al., 1993; Briscoe and Wilkinson, 2004; Philippidou and Dasen, 2013).

A large body of work in the mouse hindbrain and spinal cord has uncovered critical roles for Hox genes in defining segment identity and establishing spatial gene expression patterns necessary for neuronal differentiation during early embryogenesis (Narita and Rijli, 2009; Di Bonito et al., 2013a; Philippidou and Dasen, 2013; Parker and Krumlauf, 2020). These early Hox roles appear conserved in the zebrafish nervous system as well (Ghosh and Sagerstrom, 2018). The expression of Hox genes in the mouse hindbrain between embryonic day 7.5 (E7.5) to E9.5 appears strictly restricted within territories defined by rhombomere (transiently divided segments of the developing neural tube) boundaries (Figure 3B). Rhombomere boundaries create a series of anterior limits for Hox gene expression along the A-P axis. In the hindbrain, *Hox1-2* genes have more anterior boundaries compared to *Hox3-4* genes. On the other hand, *Hox5-13* genes are mainly expressed in the spinal cord, which is posterior to the hindbrain. The overall map of Hox gene expression along the A-P axis therefore displays spatial collinearity (Figures 3A,B). With a number of exceptions (discussed below), most Hox studies in the mouse hindbrain and spinal cord have focused on early steps of neuronal development, and thereby uncovered crucial roles for Hox in progenitor and neuronal cell fate specification, cell migration, neuronal survival, as well as axo-dendritic growth and pathfinding (Narita and Rijli, 2009; Di Bonito et al., 2013a; Philippidou and Dasen, 2013; Parker and Krumlauf, 2020).

Although Hox gene expression is well documented in early embryonic stages, their expression in late embryonic and postnatal stages is poorly characterized. Interestingly, a number of studies in the mouse hindbrain showed that the segmental Hox gene expression pattern in postmitotic neurons is also maintained in late embryonic and early postnatal stages (Pasqualetti et al., 2007; Geisen et al., 2008; Di Bonito et al., 2013b; Karmakar et al., 2017; Lizen et al., 2017a). Two systematic expression studies on the 39 mouse Hox genes revealed that the majority of Hox genes remain expressed in the hindbrain after birth and until adulthood (Hutlet et al., 2016; Tomas-Roca et al., 2016). Hutlet et al. (2016) found that the 24 Hox genes that are normally active during early development of the hindbrain continue to be expressed during adulthood. Neuroanatomical localization analysis revealed that these Hox genes are still expressed in adult post-mitotic neurons derived from rhombomeres, with visible anterior boundaries restricting individual Hox genes along the A-P axis. This indicates that the spatial collinearity rule is also maintained in adult hindbrain. Intriguingly, transcripts of some Hox genes were also identified in more anterior regions (forebrain) where they are not expressed during embryogenesis, suggesting Hox gene neo-expression in the adult CNS. More specifically, *Hoxb1, Hoxb3, Hoxb4, Hoxd3*, and *Hoxa5* transcripts were detected in both neocortex and the thalamus. Temporal analysis showed that their expression starts as early as the second postnatal week but becomes more robust only in the third postnatal week. In a separate study, Coughlan et al. have reported that the expression of *Hox9-11* genes is maintained and remains robust in spinocerebellar neurons until P7 (Coughlan et al., 2019), that is weeks after neuronal progenitor specification occurs. Of note, analysis of HOX expression in human samples showed that 15 genes are expressed in the adult brain (Takahashi et al., 2004). As in the hindbrain, Hox gene expression in the mouse embryonic spinal cord has been detected in progenitor cells and postmitotic neurons (Dasen et al., 2003, 2005; Dasen, 2009; Sweeney et al., 2018; Baek et al., 2019). A recent study focused on the brachial domain of the spinal cord found that *Hox4-8* expression is maintained in postmitotic neurons during early postnatal stages (Catela et al., 2021).

The maintained Hox expression in the mouse hindbrain and spinal cord prompts the question of what are the biological functions of mouse Hox genes in post-mitotic neurons during late developmental and postnatal stages? Below, we highlight studies on the role of mouse Hox genes in synapse formation/maturation and neuronal terminal identity; these late-occurring processes critically determine the functionality of neural circuits located in the hindbrain and spinal cord (Table 1).

### Control of Synapse Formation/Maturation by Mouse Hox Genes

The expression of Hox genes in neuronal progenitors and postmitotic neurons necessitates the employment of conditional and temporally controlled gene inactivation strategies to discriminate between early and late Hox gene functions.

The first temporally controlled Hox gene inactivation study was conducted in the trigeminal system, which relays somatosensory stimuli (e.g., touch, pain) from the face to the cortex. A key structure for such relay is the principal trigeminal nucleus in the hindbrain. Oury et al. (2006) used a tamoxifen-inducible Cre/loxP strategy to inactivate *Hoxa2* at different developmental stages in postmitotic neurons of the principal trigeminal nucleus. The authors found that late removal of *Hoxa2* leads to topographic connectivity defects of these neurons. Consistently, *Hoxa2* ectopic expression experiments suggested that maintained *Hoxa2* expression is sufficient to direct topographic axon targeting and synaptic specificity defects, potentially implicating *Hoxa2* in the regulation of molecules acting at the presynapse (Bechara et al., 2015; Gofflot and Lizen, 2018).

Apart from its role in the trigeminal system, *Hoxa2* is involved in synaptic refinement of connectivity within the brainstem auditory circuit (Karmakar et al., 2017). *Hoxa2* and *Hoxb2* are expressed throughout embryonic and postnatal life (at least up to 2 months of age) in neurons of the anterior ventral cochlear nucleus (AVCN) (Narita and Rijli, 2009). In wild-type mice, glutamatergic neurons in the AVCN, called “Bushy cells,” receive a single axonal input from one spiral ganglion neuron that forms a unique and large synapse, the endbulb of Held (Gofflot and Lizen, 2018). In mice lacking *Hoxa2* and *Hoxb2* gene activity specifically in postmitotic AVCN Bushy cells, multiple receiving inputs were observed, suggesting an involvement for these Hox genes in synapse (endbulb of Held) elimination/maturation (Karmakar et al., 2017). Importantly, these connectivity defects resulted in behavioral defects (failure to discriminate two close pure-tone frequencies) (Karmakar et al., 2017). At the molecular level, a comparative transcriptomic analysis revealed *Wnt3a* and multiple cadherins (*Cdh4, Cdh11, Cdh13, Cdh7*) as downstream targets of HOXA2/HOXB2 in AVNC Bushy cells. Given the prominent role of WNT signaling and cadherins in synapse formation and maintenance (Dickins and Salinas, 2013; Basu et al., 2015), these downstream targets could at least partially explain the connectivity defects observed in Bushy cells of mice lacking *Hoxa2* and *Hoxb2.*

In the mouse brainstem, *Hoxa5* is continuously expressed from embryonic to adult stages (Lizen et al., 2017a), suggesting its involvement at different stages of neuronal development. To test whether *Hoxa5* is functionally required in brainstem neurons, Lizen et al. (2017b) used an inducible Cre/loxP approach to inactivate *Hoxa5* at postnatal days 1–4 (P1–4) and then conducted an unbiased transcriptomic (RNA-Seq) analysis. Because *Hoxa5* expression is enriched in brainstem neurons that belong to the precerebellar system, called “pontine neurons,” it is likely this RNA-Seq approach primarily uncovered changes in RNA expression in these neurons. This study identified several genes with known roles in synapse formation and maturation as Hoxa5 targets, such as the secreted molecules *Wnt7a* and GDF10 (member of TGFβ superfamily) and the cell adhesion molecules *Cdh15* and *Fat2* (Gofflot and Lizen, 2018; Figure 3C). Consistent with these observations, a more recent study found that postmitotic *Hoxa5* expression specifies pontine neuron connectivity (Maheshwari et al., 2020).

Similar to their roles in the *C. elegans* and *Drosophila* nervous systems, mouse Hox genes can affect neural circuit formation in various ways by acting at different stages. Besides controlling synapse formation and specificity, they can also regulate axonal pathfinding which eventually leads to a failure to establish a functional neural circuit. Supporting this possibility, several mouse studies have shown that correct expression of guidance cue receptors is often co-regulated by Hox genes (Oury et al., 2006; Geisen et al., 2008; Di Bonito et al., 2013a; Maheshwari et al., 2020). For example, in the precerebellar anterior extramural migrating stream, *Hox5* genes repress the repulsive Netrin receptor *Unc5b*, while *Hox2* genes positively regulate it (Di Meglio et al., 2013). Moreover, *Hoxa2* is required for the expression of Slit receptor *Robo3* in commissural neurons in the hindbrain and *Robo2* in precerebellar pontine neurons (Di Bonito et al., 2013a).

In spinal cord circuits, several studies revealed connectivity defects upon Hox gene inactivation (Dasen et al., 2005, 2008; Catela et al., 2015, 2016; Baek et al., 2019). For example, *Hox5* genes are required for proper connectivity of phrenic motor neurons to premotor interneurons and the diaphragm muscle (Philippidou et al., 2012; Vagnozzi et al., 2020). The phrenic motor neurons express a unique combination *Hox5*-dependent cell adhesion molecules of the Cadherin (Cdh) family (Vagnozzi et al., 2020), which is known to control neuronal connectivity across model systems. Importantly, early or late genetic removal of *Hox5* in mice affects diaphragm innervation, suggesting a continuous Hox requirement for establishment and maintenance of neuronal wiring (Philippidou et al., 2012).

Conditional inactivation studies of *Hoxc8* also revealed striking connectivity defects in spinal neurons. That is, *Hoxc8* removal specifically in sensory neurons affects sensory-motor connectivity (Shin et al., 2020), whereas motor neuron-specific depletion of *Hoxc8* affects forelimb muscle innervation (Catela et al., 2016). Mechanistically, *Hoxc8* controls expression of axon molecules *Ret* and Gfrα to establish proper muscle innervation (Figure 3D). Besides their role in axon guidance, many of the aforementioned axon guidance molecules are also required for synapse formation and plasticity. This leads to the possibility that Hox genes may also maintain synaptic plasticity at post-natal stages, as suggested by the aforementioned Hoxa5 study in pontine neurons (Lizen et al., 2017b).

### Control of Neuronal Terminal Identity by Mouse Hox Genes

Recent work suggests that mouse Hox genes, similar to their *C. elegans* and *Drosophila* counterparts, control terminal identity features (e.g., NT biosynthesis components, NT receptors, ion channels) of post-mitotic neurons. In the context of pontine neurons in the brainstem, *Hoxa5* appears necessary for the maintained expression of genes encoding several glutamate receptor subunits (*Grm4, Grin2c, Grid2*), which are required for glutamatergic input by pyramidal cells (Lizen et al., 2017b). Moreover, *Hoxa5* also ensures the maintained expression of *Slc17a7* (VGLUT1), which is crucial for loading synaptic vesicles with glutamate – a key step for the synaptic output of pontine neurons onto granule cells of the cerebellum (Lizen et al., 2017b; Gofflot and Lizen, 2018; Figure 3C). In the mouse spinal cord, *Hoxc8* is required for the induction and maintenance of several terminal identity genes (*Nrg1, Mcam*, *Pappa*) in motor neurons of the brachial region (Catela et al., 2021; Figure 3D). Interestingly, while these terminal differentiation genes require Hoxc8 for both initiation and maintenance of their expression, not all Hoxc8 target genes behave in the same way. In fact, the suite of Hoxc8 targets in brachial MNs is dynamic across different life stages. For example, the glycine receptor subunit alpha-2 (*Glra2*) appears significantly downregulated upon conditional knockout of *Hoxc8* at postnatal day 8 (p8) but is unaffected upon *Hoxc8* knockout at embryonic day 12 (e12). One possible explanation for this phenomenon is that *Glra2* is redundantly regulated by additional transcription factors at early stages, whose expression fades away later on and *Hoxc8*-mediated regulation becomes necessary for maintenance. Although the underlying mechanisms remain elusive, these findings suggest that Hox genes are continuously required in the mouse nervous system to establish and maintain neuronal terminal identity features.

## Conclusion

A large body of work has uncovered critical roles for Hox genes in the early steps of nervous system development, such as progenitor cell specification, neuronal migration, cell survival and axo-dendritic growth. This review highlights recent studies in *C. elegans, Drosophila*, and mice that identified later functions for Hox genes in post-mitotic neurons, such as the control of synapse formation/maturation and neuronal terminal identity. These studies strongly suggest that Hox proteins multitask over time within a neuronal lineage by acting at the level of progenitors and/or post-mitotic neurons. Precisely controlled, temporal inactivation of Hox gene activity is necessary to continue uncovering the breadth of Hox gene functions in the nervous system. The realization of this ambitious goal critically relies on inducible genetic approaches coupled with powerful transcriptomic, biochemical, and behavioral methods.

## Author Contributions

WF and YL: literature search, writing — original draft, and review and editing. PK: supervision, literature search, writing — original draft, and review and editing. All authors contributed to the article and approved the submitted version.

## Conflict of Interest

The authors declare that the research was conducted in the absence of any commercial or financial relationships that could be construed as a potential conflict of interest.

## Publisher’s Note

All claims expressed in this article are solely those of the authors and do not necessarily represent those of their affiliated organizations, or those of the publisher, the editors and the reviewers. Any product that may be evaluated in this article, or claim that may be made by its manufacturer, is not guaranteed or endorsed by the publisher.
